# Does myo‐inositol supplementation influence oxidative stress biomarkers in patients with non‐alcoholic fatty liver disease?

**DOI:** 10.1002/fsn3.3842

**Published:** 2023-12-06

**Authors:** Somayeh Rostami, Sara Arefhosseini, Helda Tutunchi, Manouchehr Khoshbaten, Mehrangiz Ebrahimi‐Mameghani

**Affiliations:** ^1^ Student Research Committee, Faculty of Nutrition & Food Sciences Tabriz University of Medical Sciences Tabriz Iran; ^2^ Endocrine Research Center Tabriz University of Medical Sciences Tabriz Iran; ^3^ Department of Internal Medicine, Faculty of Medicine Tabriz University of Medical Sciences Tabriz Iran; ^4^ Nutrition Research Center, Department of Biochemistry and Diet Therapy, Faculty of Nutrition & Food Sciences Tabriz University of Medical Sciences Tabriz Iran

**Keywords:** myo‐inositol (MI), non‐alcoholic fatty liver disease (NAFLD), obesity, oxidative stress

## Abstract

Myo‐inositol (MI) is a carbocyclic sugar polyalcohol. MI has known to exert anti‐inflammatory, anti‐oxidant, and anti‐diabetic activities. This study aimed to investigate the effects of MI supplementation on oxidative stress biomarkers in obese patients with non‐alcoholic fatty liver disease (NAFLD). In this double‐blinded placebo‐controlled randomized clinical trial, 51 newly diagnosed obese patients with NAFLD were randomly assigned to receive either MI (4 g/day) or placebo supplements accompanied by dietary recommendations for 8 weeks. Oxidative stress biomarkers, nutritional status, as well as liver enzymes and obesity indices were assessed pre‐ and post‐intervention. A total of 48 patients completed the trial. Although anthropometric measures and obesity indices decreased significantly in both groups, the between‐group differences adjusted for confounders were non‐significant for these parameters, except for weight (*p* = .049); greater decrease was observed in the MI group. Iron and zinc intakes decreased significantly in both groups; however, between‐group differences were non‐significant at the end of the study. No significant between‐group differences were revealed for other antioxidant micronutrients at the study endpoint. Sense of hunger, feeling to eat, desire to eat sweet and fatty foods reduced significantly in both groups (*p* < .05), while the feeling of satiety increased significantly in the placebo group (*p* = .002). No significant between‐group differences were observed for these parameters, except for desire to eat fatty foods; a greater decrease was observed in the MI group (*p* = .034). Serum levels of glutathione peroxidase (GPx) and superoxide dismutase (SOD) significantly increased in both study groups (*p* < .05); however, the between‐group differences were non‐significant at the end of the study. Furthermore, the between‐group differences were non‐significant for other oxidative stress biomarkers, except for serum nitric oxide (NO) level; a greater decrease was observed in the MI group. MI supplementation could significantly improve weight, desire to eat fatty foods, serum levels of NO, as well as the aspartate aminotransferase (AST)/ALT ratio.

## Graphical abstract

This study aimed to investigate the effects of myo‐inositol (MI) supplementation on oxidative stress biomarkers in obese patients with NAFLD. MI supplement could significantly improve weight, desire to eat fatty foods, serum levels of alanine aminotransferase (ALT) and NO, as well as the aspartate aminotransferase (AST)/ALT ratio.

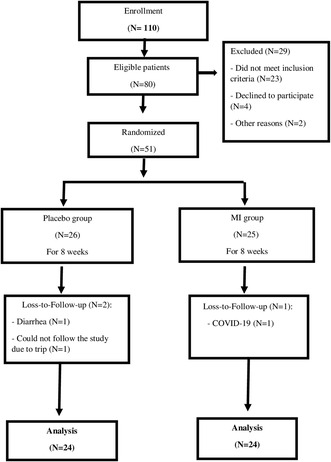

## 1 INTRODUCTION

Non‐alcoholic fatty liver disease (NAFLD) is one of the most common chronic liver diseases (Bugianesi et al., 2002). NAFLD encompasses a spectrum of liver diseases, including simple steatosis, non‐alcoholic steatohepatitis (NASH), fibrosis, and cirrhosis. Currently, NAFLD is the hepatic manifestation of insulin resistance (IR) and metabolic syndrome (Méndez‐Sánchez et al., 2007). In 2021, the prevalence of NAFLD was estimated at 32.4% worldwide, roughly 46.9 cases per 1000 person‐years. Moreover, NAFLD affected 31% of the general population in Asia and 33.9% in Iran in 2018 (Moghaddasifar et al., 2016). NAFLD affects about 8%–45% of obese adults worldwide, and the prevalence is increasing in European and Asian countries due to the increased prevalence of obesity, type 2 diabetes (T2DM), and metabolic syndrome (Tutunchi et al., 2020).

“Multiple‐hit hypothesis” has been proposed for the pathogenesis of NAFLD. In the first hit, IR results in the accumulation of triglycerides in hepatocytes, changes in the intestinal microbiome, alterations in the production and secretion of adipokines and inflammatory cytokines (Tilg et al., 2021). Hormones and enzymes, lifestyle dietary habits, and environmental factors, as the next hits, play a crucial role in the pathogenesis of NAFLD (Buzzetti et al., 2016). As a consequence, lipid accumulation in the liver disrupts the mitochondrial electron transport chain, increases the production of reactive oxygen species (ROS) and activates endoplasmic reticulum stress‐associated mechanisms (Arab et al., 2018). Therefore, oxidative stress (OS) and inflammation lead to disturbed oxidative phosphorylation, followed by a reduction in hepatic ATP synthesis and apoptosis resulting from (or due to) caspases (Farzanegi et al., 2019). Mitochondrial dysfunction disrupts the balance between pro‐oxidant and antioxidant factors, disturbs beta‐oxidation reactions, and consequently contributes to the accumulation of triglycerides in the liver, leading to obesity and IR (Masarone et al., 2018; Tilg et al., 2021).

Nowadays, the modulation of OS is one of the most important therapeutic strategies associated with lifestyle modification. Antioxidants are proposed as a promising approach for NAFLD treatment due to their effects on lipogenesis, fat oxidation, peroxidation, and inflammation (Buzzetti et al., 2016). The conventional therapeutic approaches for NAFLD recommended by the leading research and medical societies are a calorie‐restricted diet and increased physical activity due to their effects on insulin sensitivity as well as liver fat and enzymes (Benedict & Zhang, 2017; Day & James OF, 1998; Masarone et al., 2018; Tutunchi et al., 2019). Therefore, antioxidants have been suggested as a new solution for patients with NAFLD, as antioxidants influence lipogenesis, fat oxidation, peroxidation, and inflammation. Previous studies have shown that OS stimulates liver fibrosis. This reaction is carried out by increasing pro‐inflammatory cytokines and activating hepatic stellate cells (HSCs) (Tang et al., 2021). Abenavoli et al. support the benefit of antioxidant supplementation in patients with NAFLD by studying 50 overweight patients (Abenavoli et al., 2017). Federico et al found that consumption of antioxidants (303 mg silybin‐phospholipid complex, 10 mg vitamin D, and 15 mg vitamin E) twice daily for 6 months led to significant improvements in alanine transaminase (ALT) and gamma glutamyl transpeptidase (γGT) levels in patients with NAFLD compared to the healthy controls (Federico et al., 2019). Recently, the therapeutic role of inositol (INS) in NAFLD has received more attention. Pinitol – another inositol derivative – has been shown to reduce liver fat, triglycerides, aspartate aminotransferase (AST) and lipid peroxidation, and increase glutathione peroxidase (GPx) activity (Pani et al., 2020). The common form of INS derivatives is myo‐inositol (MI), which is naturally isolated from cereals (e.g., rice bran) and stored in the liver, kidney, and brain. It can also be synthesized from D‐glucose in the body. MI is a cyclic alcohol that plays a role in fundamental reactions in the body, including signal transduction, insulin signaling, and mediating calcium regulation in membrane signaling. Moreover, MI has been demonstrated to exert anti‐cancer, anti‐inflammatory, and anti‐oxidant activities (Bradford et al., 2017; Kiani et al., 2021). MI increases the activity of the antioxidant system by elevating the activity of glucose in the pentose phosphate pathway to produce more NADPH (Rushworth et al., 2006). There is evidence indicating that MI activates protein kinase C, increases NF‐E2–related factor 2 (Nrf2) in monocytes, and improves antioxidant enzymes from this pathway (Jiang et al., 2013; Wang et al., 2010).

In the study on rats suffering from hepatotoxicity, supplementation with pinitol decreased the levels of AST and ALT (Zhou et al., 2008). Furthermore, oral administration of D‐*chiro*‐inositol (DCI) (150 mg/kg) was shown to improve the severity of liver fibrosis and ALT and AST levels after cholestatic induction in rats (Zhao et al., 2018). In another study, taking MI plus folic acid among subjects diagnosed with polycystic ovary syndrome (PCOS) had beneficial effects on the level of plasma total antioxidant capacity (TAC) (Jamilian et al., 2017; Jamilian et al., 2018). In a study performed in patients with NAFLD, regular low‐dose or high‐dose pinitol supplementation significantly reduced the content of liver fat and improved the activities of GPx and malondialdehyde (MDA) enzymes (Lee et al., 2019). Since there is controversy over the findings of previous studies and also because the effect of MI supplementation on OS factors has been rarely studied in humans, particularly in NAFLD, the present clinical trial aimed to investigate the effects of MI supplementation on OS biomarkers in patients with NAFLD.

## 2 MATERIALS AND METHODS

### 2.1 Study design and patients

Male and female patients with obesity (body mass index (BMI) 30–40 kg/m^2^) aged 18–55 years who were newly diagnosed with NAFLD without taking any treatment were recruited in this double‐blind placebo‐controlled randomized clinical trial between October 2021 and March 2022. The trial protocol was approved not only by the Ethics Committee of Research vice‐chancellor of Tabriz University of Medical Sciences (Ethical code: IR.TBZMED.REC.1400.566) but also by the Iranian clinical trials registration (http://www.irct.ir; registration number: IRCT20100209003320N2). An informed consent form was signed by each patient after presenting a full explanation of the study objectives and protocol.

The diagnosis of NAFLD was conducted by a radiologist using ultrasonography (Sonoace X4 Medisio, South Korea) while the patient was fasting. The degree of liver steatosis was based on the following four parameters: hepatorenal echocontrast, liver brightness, deep attenuation, and vessel blurring, which are considered as the main determiners for the scoring system of NAFLD proposed by Hamaguchi et al (Hamaguchi et al., 2007). Based on these parameters, the grading of NAFLD was scored as 1 = mild, 2 = moderate, and 3 = severe steatosis. It should be noted that only patients with grade 1 and 2 fatty liver were included in the present study. Patients with grade 3 NAFLD, NASH, and other liver diseases such as viral or autoimmune hepatitis, drug‐induced hepatitis, cirrhosis, and biliary disorders were not included in the study. Moreover, those who were pregnant, lactating, postmenopause, athletes, smokers, or alcohol drinkers, who followed weight loss programs over the last 3 months, those with diseases such as cardiovascular, kidney, intestinal, thyroid, or parathyroid dysfunction, PCOS, cancer, malabsorption, and known autoimmune diseases, as well as those taking corticosteroids, non‐steroidal anti‐inflammatory drugs and chemical or herbal supplements for weight loss affecting liver enzymes, and vitamins over the last 3 months prior to the study were excluded.

### 2.2 Sample size

The sample size was calculated based on the mean ± standard deviation (SD) of changes in serum levels of TAC reported by Jamilian et al. (Jamilian et al., 2018). By considering a 95% confidence level and 80% power using the G power software, 21 patients for each group were estimated, which increased to 25 patients in each arm because of a 20% dropout rate.

### 2.3 Randomization, blinding, and intervention

The eligible patients were randomly assigned in a 1:1 ratio into two groups (MI or placebo) using the random allocation software (RAS) in blocks stratified by gender (male vs. female, age (20–35 vs. 36–50 years), and BMI status (30–34.9 kg/m^2^ vs. 35–39.9 kg/m^2^) by an independent person who was not involved in the study. Randomization was performed by a statistician who was blinded to the study process. The participants and researchers were blinded to study allocation until the end of the analysis.

MI powder (Wholesale Health Connection, China) as well as maltodextrin powder were packaged into 2 g sachets in a hygiene condition. The sachets were completely similar in all aspects, including appearance, size, and color. Those in *the “MI group”* or “*Placebo group*” (*n* = 25) received two 2 g sachets of MI or maltodextrin powder daily (before lunch and dinner) by dissolving in one glass of water (240 cc). MI and placebo sachets were fortnightly provided, and the patients were asked to return unused sachets to determine the compliance rate. The consumption of ≥90% of the supplements was considered “*adherence*”. Hence, healthy eating recommendations were given to all participants at the beginning of the study. The participants were asked not to change the type or duration of their physical activity.

### 2.4 Anthropometric and physical activity assessment

Pre‐ and post‐intervention, height and weight were measured in a fasting state without shoes with light clothes, using a caliper and a Seca stadiometer (Seca, Hamburg, Germany) to the nearest 0.1 cm and 0.1 kg, respectively. Waist circumference (WC) – as midway between the lowest rib and iliac blades – and hip circumference (HC) – as the widest circumference over the great trochanters – were measured using a non‐stretchable measuring tape to the nearest 0.1 cm. Then, obesity indexes, including BMI, waist‐to‐hip ratio (WHR), and waist‐to‐height ratio (WHR), were estimated.

A short form of the International Physical Activity questionnaire (IPAQ‐SF) was also completed for each patient through face‐to‐face interviews at the beginning and end of the study. The patients' responses were converted to Metabolic Equivalent Task minutes per week (MET‐min/week) and then the calculated MET score was classified into 3 levels, i.e. “*high*,” “*moderate*,” or “*low*” levels of physical activity using the metabolic equivalent task (MET‐hours/week) score based on the manual (Committee, 2005).

### 2.5 Assessment of appetite sensations and dietary intakes

Assessment of appetite sensations was performed by the Visual Analogue Scale using a reproducible and valid 6‐item questionnaire. To assess appetite, the patients were asked about the feeling of hunger, satiety, fullness, and the desire to consume something sweet, fatty, or salty using a validated questionnaire at baseline and at the end of the study (Flint et al., 2000). Moreover, dietary intake was assessed using a 3‐day food recall (two non‐consecutive weekdays and one weekend at each stage) at baseline and endpoint. Food consumption was estimated in terms of grams and milliliters as an average on a daily basis, and then energy and nutrient intakes were analyzed using Nutritionist IV (N4) software, which was modified for Iranian foods (First Databank, San Bruno, CA, USA).

### 2.6 Biochemical measurements

After 12 hours of overnight fasting, 10 mL of venous blood samples were collected from each patient at baseline and at the end of the study. Five mL of whole blood was collected in EDTA vacuum blood collection tubes to prevent blood clotting, and the rest of the 5 mL was collected in vacuumed gel separator tubes for centrifugation. The blood collected in vacuumed gel separator tubes after 20 min was centrifuged at a speed of 3000 rpm for 8 min. After serum extraction, serum samples were collected in 1 mL microtubes for storage in a −80 degree freezer to measure nitric oxide (NO), arylesterase activity linked to paraoxonase‐1 (PON1), MDA, catalase (CAT), and TAC factors. The blood collected in EDTA vacuum blood tubes was kept in a freezer at −18°C to determine the levels of GPX, CAT, and superoxide dismutase (SOD). ALT and AST were measured using the enzymatic–colorimetric method using kits (Pars‐Azmoon Co., Tehran, Iran). Serum TAC, GPx, and SOD were assessed using the spectrophotometric method and the Randox kit (Laboratories, Crumlin, UK), while serum MDA level was determined based on the reaction with thiobarbituric acid (TBA). Blood hemoglobin was also measured using a commercial kit (BioRex Co., Tehran, Iran) for estimating SOD and GPX. The paraoxonase activity of arylesterase was chemically assessed using the following formula after performing the chemical reaction:

$$\frac{A1-A2}{0.5}\times 200$$

### 2.7 Statistical analysis

SPSS statistics software (SPSS Inc., Chicago, IL, USA, version 23) was used for data analysis. Analyses were performed based on an intention‐to‐treat (ITT) approach. For checking the normality of continuous variables, the Kolmogorov–Smirnov test was used. Data were reported as mean (SD) for numerical data and frequency (percentage) for categorical variables. An independent sample *t*‐test was applied to compare variables between the two groups, while within‐group differences were tested using paired samples *t*‐ and Wilcoxon signed‐rank tests for symmetric and asymmetric continuous variables, respectively. The chi square and sign test were also used for within‐group changes for qualitative variables. Inter‐group differences at the end of the study were tested using Analysis of Covariance (ANCOVA) by adjusting for the confounders (including baseline values, weight, and energy intake). Statistical significance was defined as a *p*‐value of less than .05.

## 3 RESULTS

### 3.1 General and demographic characteristics

**FIGURE 1:**
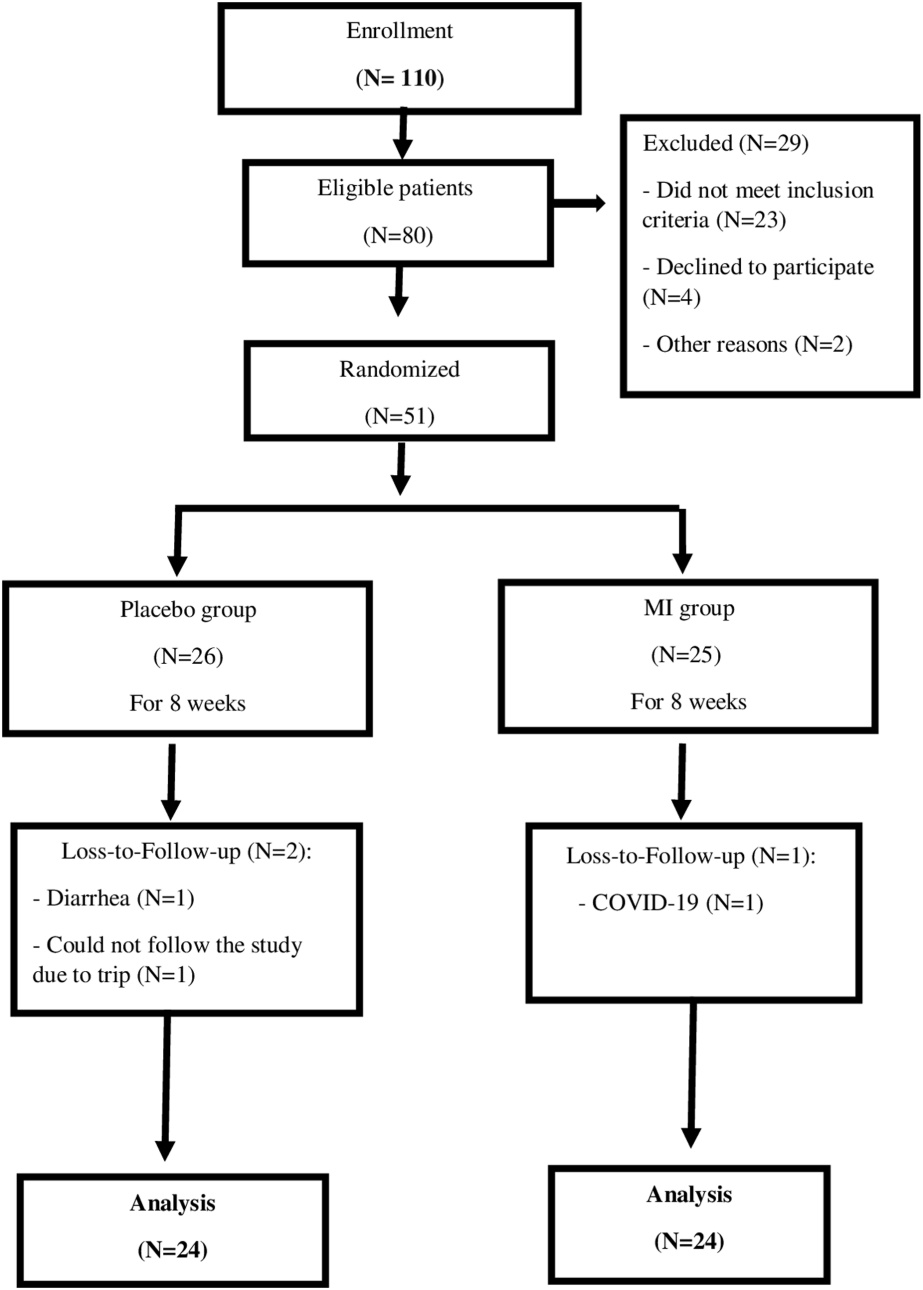
The flowchart of the study.

Of the total 51 patients enrolled, 48 patients (*N* = 24 in each group) completed the trial. Two patients in the placebo group and one patient in the MI group discontinued because of reasons unrelated to the interventions (Figure 1).

Table 1 shows the general characteristics of the patients at baseline. The mean age of the patients was 36.33 (8.49) years in the MI group and 37.50 (6.86) years in the placebo group. There were no significant differences in socio‐demographic characteristics, anthropometric measures, and NAFLD severity between the groups.

**TABLE 1:** Baseline characteristics of the study participants.

Variable	MI (*N* = 24) mean (SD)	Placebo (*N* = 24) mean (SD)	*p*
Age (yrs.)	36.33 (8.49)	37.50 (6.86)	.603^a^
Weight (kg)	96.43 (14.68)	96.93 (14.78)	.907^a^
Height (cm)	168.73 (9.6)	168.23 (10.15)	.859^a^
BMI (kg/m^2^)	33.77 (3.52)	34.16 (3.37)	.701^a^

	(%)	(%)	
Gender			
Male	13 (54.2)	11 (45.8)	.564^b^
Female	11 (45.8)	13 (54.2)	
Marital status			
Single	4 (16.7)	3 (12.5)	.683^b^
Married	20 (83.3)	21 (87.5)	
Educational level			
Up to Diploma	13 (54.2)	7 (29.2)	.079^b^
University degree	11 (45.8)	17 (70.8)	
Occupation			
Housewife	8 (33.3)	10 (41.7)	.456^b^
Student	3(12.5)	1 (4.2)	
Employee	5 (20.8)	8 (33.3)	
Self‐employed	8 (33.3)	5 (20.8)	
Fatty liver severity			
Mild	9 (37.5)	11 (45.8)	.558^b^
Moderate	13 (54.2)	15 (62.5)	

*Note*: This table has been previously published (Arefhosseini et al., 2023).

Abbreviations: BMI, Body mass index; MI, Myo‐inositol.

^a^ Independent sample *t*‐test.

^b^ Chi square.

### 3.2 Anthropometric indices, dietary intakes, and physical activity

Obesity‐related indices are demonstrated in Table 2. Although weight, BMI, WC, HC, and WHtR decreased significantly in both groups, the between‐group differences adjusted for baseline values and energy intake were non‐significant for these parameters, except for weight (*p* = .049); a greater decrease was observed in the MI group. Furthermore, there were no within‐ and between‐group differences in physical activity.

**TABLE 2:** Changes in anthropometric indices and physical activity of the study participants over the study.

	MI (*N* = 24) mean (SD)	Placebo (*N* = 24) mean (SD)	*p*
Weight (kg)			
Baseline	96.43 (14.67)	96.933 (14.78)	.907^b^
End	91.71 (14.75)	93.658 (15.16)	**.049** ^c^
MD (95% CI)	−4.72 (−6.18, −3.25)	−3.27 (−4.55, −1.99)	
*p* ^a^	**<.001**	**<.001**	
WC (cm)			
Baseline	109.854 (9.57)	111.333 (9.21)	.588^b^
End	105.646 (9.48)	106.904 (10.07)	.217^c^
MD (95% CI)	−4.20 (−5.91, −2.50)	−4.42 (−6.48, –2.36)	
*p* ^a^	**<.001**	**<.001**	
HC (cm)			
Baseline	115.667 (6.52)	117.771 (8.64)	.346^b^
End	111.812 (9.99)	115.146 (9.58)	.528^c^
MD (95% CI)	−3.85 (−4.97, −2.73)	−2.62 (−3.90, −1.34)	
*p* ^a^	**<.001**	**<.001**	
WHR			
Baseline	0.949 (0.055)	0.945 (0.048)	.820^b^
End	0.944 (0.046)	0.929 (0.058)	.129^c^
MD (95% CI)	−0.005 (−0.01, 0.007)	−0.01 (−0.03, 0.001)	
*p* ^a^	.409	.070	
WHtR			
Baseline	0.651 (0.048)	0.663 (0.057)	.444^b^
End	0.626 (0.052)	0.636 (0.058)	.225^c^
MD (95% CI)	−0.02 (−0.03, −0.01)	−0.02 (−0.03, −0.01)	
*p* ^a^	**<.001**	**<.001**	
BMI (kg/m^2^)			
Baseline	33.77 (3.53)	34.16 (3.37)	.701^b^
End	32.11 (3.55)	33 (3.64)	.052^c^
MD (95% CI)	−1.66 (−2.16, −1.16)	−1.16 (01.60, −0.72)	
*p* ^a^	**<.001**	**<.001**	
PA (METs)			
Baseline	63.53 (37.17)	57.54 (39.42)	.590^b^
End	57.69 (30.70)	54.79 (31.95)	.819^c^
MD (95% CI)	2.74 (−9.79, 15.27)	5.84 (−10.39, 22.07)	
*p* ^a^	.655	.464	

*Note*: Mean (SD) and Mean Difference (95% CI) are presented for data. Bold values indicate statistically significant (*p* < .05).

Abbreviations: BMI, Body mass index; HC, Hip circumference; METs, Metabolic equivalents (MET‐minutes/week); MI, Myo‐inositol; PA, Physical activity; WC, Waist circumference; WHR, Waist‐to‐hip ratio.

^a^ *p*‐value for paired *t*‐test.

^b^ *p*‐value for independent samples *t*‐test.

^c^ *p‐*value for ANCOVA test (adjusted for baseline values and energy intake).

Table 3 demonstrates changes in dietary intakes of antioxidant micronutrients in both groups over course of the study. Iron and zinc intakes decreased significantly in both groups; between‐group differences were non‐significant by the end of the study, though. Moreover, no significant between‐group differences were revealed for other antioxidant micronutrients at the study endpoint.

**TABLE 3:** Dietary intakes of antioxidant micronutrients of the study participants throughout the study.

	MI (*N* = 24)	Placebo (*N* = 24)	*p*
Fe/iron (mg)			
Baseline	16.292 (7.79)	14.687 (5.64)	.418^b^
End	11.700 (7.64)	9.825 (2.92)	.446^c^
MD (95% CI)	−4.59 (−7.49, −1.68)	−4.86 (−6.76, −2.96)	
*p* ^a^	**.003**	**.000**	
Zinc (mg)			
Baseline	7.443 (3.5)	7.343 (2.84)	.915^b^
End	5.680 (2.89)	5.419 (2.86)	.893^c^
MD (95% CI)	−1.76 (−3.10, −0.41)	−1.92 (−3.27, −0.41)	
*p* ^a^	**.012**	**.007**	
Selenium (μg)			
Baseline	0.067 (0.037)	0.068 (0.037)	.950^b^
End	0.062 (0.042)	0.057 (0.3)	.352^c^
MD (95% CI)	−0.005 (−0.03, 0.01)	−0.01 (−0.03, −0.006)	
*p* ^a^	.554	.194	
Vitamin A			
Baseline	1684.3 (1297.8)	1159.3 (807.63)	.187^b^
End	1463.8 (1575.56)	1114.5 (1305.67)	.325^c^
MD (95% CI)	−22.04 (−1084.55, 646.59)	−44.76 (−666.26, 576.74)	
*p* ^a^	.604	.883	
Vitamin E			
Baseline	3.613 (3.01)	2.943 (1.93)	.529^b^
End	5.2179 (6.29)	3.159 (3.9)	.137^c^
MD (95% CI)	1.60 (−1.16, 4.37)	0.21 (−1.55, 1.99)	
*p* ^a^	.243	.803	
Vitamin B9			
Baseline	325.32 (174)	251.31 (92.51)	.074^b^
End	263 (212.43)	209.43 (92.28)	.526^c^
MD (95% CI)	−61.74 (−166.91, 43.43)	−41.89 (−91.46, 7.70)	
*p* ^a^	.237	.094	
Vitamin C			
Baseline	105.73 (45.23)	97.07 (57.07)	.563^b^
End	92.91 (68.82)	111.31 (69.14)	.089^c^
MD (95% CI)	−12.81 (−41.64, 16.01)	−14.23 (−10.56, 39.03)	
*p* ^a^	.367	.247	

*Note*: Bold values indicate statistically significant (*p* < .05).

Abbreviation: MI, myo‐inositol.

^a^ *p*‐value for paired *t*‐test.

^b^ *p*‐value for independent samples *t*‐test.

^c^ *p‐*value for ANCOVA test (adjusted for baseline values and energy intake).

### 3.3 Appetite sensations

The effects of MI supplementation on appetite sensations are illustrated in Table 4. Sense of hunger, feeling to eat, desire to eat sweet and fatty foods reduced significantly in both groups (*p* < .05), while the feeling of satiety increased significantly in the placebo group (*p* = .002). No significant between‐group differences were observed for these parameters, except for desire to eat fatty foods; a greater decrease was observed in the MI group (*p* = .034).

**TABLE 4:** Appetite sensations of the study participants throughout the study.

	MI (*N* = 24)	Placebo (*n* = 24)	*p*
Hungry			
Baseline	5.71 (1.94)	5.42 (2.36)	.642
End	3.79 (2.08)	3.79 (1.67)	.754
MD (95% CI)	−1.91 (−2.98, −0.84)	−1.62 (−2.77, −0.47)	
*p* ^a^	**.001**	**.008**	
Fullness			
Baseline	5.21 (1.5)	4.96 (2.44)	.671^b^
End	6.04 (2.05)	6.79 (1.79)	.176^b^
MD (95% CI)	0.83 (−0.35, 2.01)	1.83 (0.72, 2.94)	
*p* ^a^	.159	**.002**	
Feeling to eat			
Baseline	7.29 (1.56)	6.75 (2.5)	.426^b^
End	4.17 (2.18)	5.08 (2.41)	.179^b^
MD (95% CI)	−3.12 (−4.46, −1.78)	−1.66 (−2.85, −0.48)	
*p* ^a^	**<.001**	**.008**	
Desire to eat sweet foods			
Baseline	6.50 (3.03)	5.17 (2.49)	.103^b^
End	4.29 (2.79)	3.96 (2.03)	.968^b^
MD (95% CI)	−2.20 (−3.50, −0.90)	−1.20 (−2.10, −0.31)	
*p* ^a^	**.002**	**.011**	
Desire to eat salty foods			
Baseline	2.71 (2.42)	3.71 (2.66)	.180^b^
End	1.79 (2)	2.88 (1.77)	.228^b^
MD (95% CI)	−0.91 (−1.93, 0.10)	−0.83 (−1.67, 0.008)	
*p* ^a^	.075	.052	
Desire to eat fatty foods			
Baseline	4.88 (2.33)	4.04 (2.68)	.256^b^
End	2.75 (1.67)	2.79 (2.18)	**.034** ^b^
MD (95% CI)	−2.12 (−3.26, −0.98)	−1.25 (−2.38, −0.11)	
*p* ^a^	**.001**	**.032**	

*Note*: Bold values indicate statistically significant (*p* < .05).

Abbreviation: MI, myo‐inositol.

^a^ *p*‐value for paired *t*‐test.

^b^ *p*‐value for independent samples *t*‐test.

^c^ *p‐*value for ANCOVA test (adjusted for baseline values and energy intake).

### 3.4 Liver enzymes

Table 5 demonstrates changes in serum liver enzymes. As we reported in our previously published paper (Arefhosseini et al., 2023), serum levels of ALT and AST decreased significantly in both groups. At the end of the study, MI supplementation for 8 weeks only showed improvements in the AST/ALT ratio but not in serum AST, after adjusting for the confounders. Moreover, MI supplementation was clinically effective in reducing the severity of liver steatosis. The estimated number needed to treat (NNT) for a one‐grade reduction in steatosis severity was three patients, while the NNT for a two‐grade reduction was eight patients. In other words, of every three and eight patients with NAFLD who took MI (at a dosage of 4 g/day) for 8 weeks, one patient would experience a one‐grade reduction in liver steatosis and treat completely (two‐grade reduction), respectively.

**TABLE 5:** Liver parameters of the study participants throughout the study.

	MI (*N* = 24)	Placebo (*n* = 24)	*p*
ALT (IU/L)			
Baseline	37.42 (23.36)	42.83 (30.99)	.498^b^
End	25.21 (10.10)	28.56 (13.19)	**.027** ^c^
MD (95% CI)	−12.21 (−20.44, −3.99)	−14.27 (−24.40, −4.14)	
*p* ^a^	**.005**	**.008**	
AST			
Baseline	31.41 (16.53)	28.50 (13.05)	.501^b^
End	19.69 (4.30)	21.38 (6.49)	.066^c^
MD (95% CI)	−11.72 (−17.90, −5.54)	−7.12 (−11.81, −2.44)	
*p* ^a^	**.001**	**.005**	
AST/ALT ratio			
Baseline	0.993 (0.4)	0.77 (0.24)	.024^b^
End	0.871 (0.28)	0.819 (0.24)	**.006** ^c^
MD (95% CI)	0.12 (−0.25, 0.01)	0.50 (−0.03, 0.14)	
*p* ^a^	.069	.259	

*Note*: Mean (SD) and mean difference (95% CI) are presented for data. Bold values indicate statistically significant (*p* < .05). This table has been previously published (Arefhosseini et al., 2023).

Abbreviations: ALT, alanine transaminase; AST, aspartate aminotransferase; AST/ALT, AST‐to‐ALT ratio; MI, myo‐inositol.

^a^ *p*‐value for paired *t*‐test.

^b^ *p*‐value for independent samples *t*‐test.

^c^ *p‐*value for ANCOVA test (adjusted for baseline values and energy intake).

### 3.5 Antioxidant and oxidative stress markers

As presented in Table 6, serum levels of GPx and SOD significantly increased in both study groups (*p* < .05); however, the between‐group differences were non‐significant at the end of the study when adjusted for the baseline values and energy intake. The between‐group differences adjusted for potential confounders were non‐significant for other OS biomarkers, except for serum NO level; a greater decrease was observed in the MI group.

**TABLE 6:** Antioxidant and oxidative stress markers of the study participants throughout the study.

	MI (*N* = 24)	Placebo (*n* = 24)	*p*
PON1			
Baseline	36.11 (6.43)	36.39 (7.71)	.893^b^
End	36.87 (6.28)	36.88 (5.13)	.926^c^
MD (95% CI)	0.76 (−2.54, 4.06)	0.48 (−3.20, 4.18)	
*p* ^a^	.638	.787	
NO			
Baseline	4.5104 (1.08)	4.923 (1.27)	.235^b^
End	4.097 (0.835)	4.790 (1.26)	**.025** ^c^
MD (95% CI)	−0.41 (−0.90, −0.06)	−0.13 (−0.74, 0.48)	
*p* ^a^	.087	.660	
CAT			
Baseline	85.603 (24.74)	74.542 (19.106)	.090^b^
End	98.850 (30.90)	82.829 (22.02)	.098^c^
MD (95% CI)	1.32 (−2.46, 28.96)	8.28 (−3.01, 19.59)	
*p* ^a^	.095	.143	
GPX			
Baseline	67.169 (8.16)	67.637 (6.37)	.826^b^
End	70.651 (8.40)	71.70 (7.33)	.917^c^
MD (95% CI)	3.48 (1.11, 5.85)	4.07 (1.13, 7.00)	
*p* ^a^	**.006**	**.009**	
SOD			
Baseline	1283.1 (178.19)	1271.6 (152.43)	.811^b^
End	1546.8 (175.91)	1551.6 (138.00)	.989^c^
MD (95% CI)	263.68 (175.54,351.82)	280.04 (181.81, 378.28)	
*p* ^a^	**<.001**	**<.001**	
MDA			
Baseline	1.765 (0.51)	1.781 (0.65)	.926^b^
End	1.842 (0.45)	1.705 (0.39)	.301^c^
MD (95% CI)	0.076 (−0.13, 0.29)	−0.076 (−0.35, 0.19)	
*p* ^a^	.469	0.569	
TAC			
Baseline	1.602 (0.28)	1.542 (0.26)	.454^b^
End	1.567 (0.20)	1.525 (0.3)	.768^c^
MD (95% CI)	−0.03 (−0.14, 0.07)	−0.17 (−0.15, 0.11)	
*p* ^a^	.521	.787	

*Note*: Mean (SD) and mean difference (95% CI) are presented for data. Bold values indicate statistically significant (*p* < .05).

Abbreviations: CAT, *Catalase*; GPX, *Glutathione peroxidase*; MDA, malondialdehyde; MI, Myo‐inositol; NO, *nitric oxide*; PON1, arylesterase activity of paraoxonase‐1; SOD, *Superoxide dismutase*; TAC, Total antioxidant capacity.

^a^ *p*‐value for paired *t*‐test.

^b^ *p*‐value for independent samples *t*‐test.

^c^ *p‐*value for ANCOVA test (adjusted for baseline values and energy intake).

## 4 DISCUSSION

The results of the present clinical trial investigating the effect of MI supplementation on OS in patients with NAFLD showed that MI supplementation for 8 weeks could significantly influence weight, desire to eat fatty foods, and NO. The feeling of hunger, the desire to eat food, and the desire to eat sweet and fatty foods were significantly reduced in both groups. Apart from weight, MI supplementation compared to placebo did not affect not only anthropometric measures but also obesity indices at the end of the study (Table 2). This finding is consistent with the results of previous studies (Fruzzetti et al., 2017; Pintaudi et al., 2016). For example, Pintaudi et al. failed to find any effect of 1100 mg of MI supplementation on BMI in adults with T2DM (Pintaudi et al., 2016) while Pkhaladze et al. reported a significant reduction in weight and BMI in adolescents after 3 months (Pkhaladze et al., 2021). Moreover, the results of a meta‐analysis including 15 controlled clinical trials showed that inositol supplementation significantly reduced BMI (Zarezadeh et al., 2022). In diabetic rats, supplementation with inositol derivatives, especially inositol hexaphosphate (IP6), resulted in weight reduction through increasing serum leptin, which in turn regulated food intake (Foster et al., 2016). Several studies have reported the effectiveness of DCI and MI in improving obesity‐related complications in patients with PCOS and metabolic syndrome (Giordano et al., 2011; Unfer et al., 2016; Zheng et al., 2015). It has also been shown that IP6 reduces blood glucose and delays carbohydrate digestion and absorption in humans. The effect is clinically small and cannot be considered as a single therapeutic approach; therefore, it is suggested to be applied accompanied by other interventions such as weight loss or energy‐restricted diets, dietary supplements, and exercise. The beneficial effect of inositol on obesity and related disorders may be explained by its involvement in insulin signaling and improving insulin sensitivity (Zarezadeh et al., 2022). However, the short duration of the trial and the difference in the type of disease in the studied population are also involved in inconsistent findings.

Our results also revealed that MI supplementation resulted in not only a significant increase in serum antioxidants, GPX, and SOD but also a significant reduction in NO biomarkers (Table 6 **)**. Our findings are consistent with other studies (Jamilian et al., 2018; Lee et al., 2019; Pan et al., 2022) reporting the effectiveness of inositol supplementation on antioxidant indices. Inositol derivatives appear to play a protective role against OS caused by cell metabolism (Bizzarri et al., 2016). Pan et al. (Pan et al., 2022) showed that dietary inositol can significantly increase the activities of SOD, CAT, GPX, and TAC contents and reduce the content of MDA as well as ROS. MI supplementation also increases the activity of glucose in the pentose phosphate pathway through more production of NADPH, increasing the ratio of NADPH to NADP^+^ and the activity of the antioxidant system, and maintaining cell health. It should be emphasized that NADPH is one of the factors that reduce stress in cells and is an essential factor for the normal functioning of antioxidant cycles such as glutathione thioredoxin systems (Jiang et al., 2014). Protein kinase C (PKC) plays an important role in Nrf2 activation in human monocytes. MI signaling may activate PKC; therefore, the increase of MI plays a role in the activity of the antioxidant system through the PKC/Nrf2 pathways (Jiang et al., 2013). Nevertheless, Jamilian et al. reported that MI (2 g) plus folic acid (200 μg) supplementation twice a day compared to 500 mg metformin (3 times/day) for 12 weeks had no effect on plasma NO levels in patients with PCOS. It appears that factors such as the lower dosage of the supplement, simultaneous use of MI with folic acid, or the type of disease of the study group could be involved in obtaining the different results.

MI supplementation improved liver enzymes, which is consistent with the results of the studies conducted by Zhao et al. (Zhao et al., 2018), Lee et al. (Lee et al., 2019), and also Pan et al. (Pan et al., 2022). Moreover, we found that MI supplementation for 8 weeks could reduce at least one degree of liver steatosis severity in patients with NAFLD, which is in line with the results of an animal study conducted by Zhao et al. after DCI supplementation (150 mg/kg for 2 weeks) in rats (Zhao et al., 2018). Zhu et al. (Zhou et al., 2008) also showed that pinitol supplementation suppressed increased ALT and AST in rats fed a high‐fat diet with liver damage. We also reported significant improvement in not only IR and lipid profiles but also liver function in patients with NAFLD (Arefhosseini et al., 2023). Genetic factors with great effect on IR (as a focal effect of NAFLD) and liver enzymes cause a disturbance in the normal function of the liver, which not only increases ALT and AST as the most specific factors, but also gamma glutamyl transferase (GGT), bilirubin, and ferritin (Croze & Soulage, 2013; Tabrizi et al., 2018). The insulin mimetic property of MI supplementation increases insulin sensitivity and has a positive effect on metabolic disorders, gene expression, inflammatory pathways, OS biomarkers, and hormonal states (Chatree et al., 2020; Croze & Soulage, 2013; Tabrizi et al., 2018). Therefore, MI appears to be a powerful element in NAFLD management.

The present study had some limitations, such as a short duration, a relatively small sample size, and studying only obese patients with NAFLD. In addition, although liver biopsy is currently the gold standard for diagnosing NAFLD, it could not be done in the current study due to ethical considerations and participants' refusal. Our study had some strengths too. A major strength of this study was that it could be counted as the first human trial investigating the efficacy of MI supplementation in conjunction with dietary recommendations on OS biomarkers and liver function in newly diagnosed obese patients with NAFLD. Moreover, consideration of confounding variables, using a placebo, and patients’ follow‐up are considered strengths.

## 5 CONCLUSION

MI supplement could significantly improve weight, desire to eat fatty foods and the AST/ALT ratio. Serum levels of GPx and SOD significantly increased in both study groups; however, the between‐group differences were non‐significant at the end of the study. Furthermore, the between‐group differences were non‐significant for other OS biomarkers, except for serum NO level; a greater decrease was observed in the MI group. Further clinical trials with larger sample sizes and longer durations are encouraged to study the different doses of MI and other INS derivatives in patients with NAFLD.

## AUTHOR CONTRIBUTIONS

**Somayeh Rostami:** Investigation (equal); methodology (equal); project administration (equal); writing – original draft (equal). **Sara Arefhosseini:** Investigation (equal); methodology (equal); project administration (equal). **Helda Tutunchi:** Conceptualization (equal); writing – review and editing (equal). **Manouchehr Khoshbaten:** Visualization (equal). **Mehrangiz Ebrahimi‐Mameghani:** Conceptualization (equal); funding acquisition (equal); resources (equal); supervision (equal); validation (equal); visualization (equal).

## FUNDING INFORMATION

This study was funded by the ‘Research Vice‐Chancellor’ of Tabriz University of Medical Sciences, Tabriz, Iran. This paper is a part of the data obtained from an MSc dissertation submitted to Tabriz University of Medical Sciences (Somayeh Rostami).

## CONFLICT OF INTEREST STATEMENT

The authors affirm that there were no conflict of interest.

## ETHICS STATEMENT AND CONSENT TO PARTICIPATE

All procedures performed in this study were in accordance with the ethical standards of the Ethics Committee of Tabriz University of Medical Science. In this study, all subjects signed a consent form and the study protocol was approved by the Ethical Committee of Tabriz University of Medical Sciences (Ethics code: TBZMED. REC.1400.566).

## Data Availability

The datasets used and/or analyzed during the current study are available from the corresponding author on a reasonable request.
